# A parafoveal retinal cones analysis using adaptive-optics retinal camera in patients with primary open angle glaucoma

**DOI:** 10.1038/s41433-024-03191-1

**Published:** 2024-09-02

**Authors:** Eleonora Trolli, Matilde Roda, Nicola Valsecchi, Davide Cacciatore, Elena Nardi, Valentina Della Pasqua, Andrea Mercanti, Luigi Fontana

**Affiliations:** 1 Ophthalmology Unit, DIMEC, Alma Mater Studiorum Università di Bologna, Bologna, Italy; 2 IRCCS Azienda Ospedaliero-Universitaria di Bologna, Bologna, Italy; 3 Ophthalmology Unit, Head and Neck Surgical Department of Ophthalmology, Infermi Hospital, Rimini, Italy; 4 Research and Innovation Unit, IRCCS Azienda Ospedaliero-Universitaria di Bologna, Bologna, Italy

**Keywords:** Glaucoma, Optic nerve diseases

## Abstract

**Objectives:**

To study the density, spacing, and regularity of retinal cone photoreceptors using an Adaptive Optics (AO) retinal camera (Rtx1^TM^, Imagine Eyes, Orsay, France) in patients with Primary Open Angle Glaucoma (POAG) and to compare the outcomes with those of healthy age-matched control subjects.

**Methods:**

The study included 43 eyes with POAG and 31 eyes of normal subjects. POAG patients were divided into three groups according to the severity of the visual field defect. The AO Rtx1^TM^ was used to obtain images of the parafoveal cone mosaic to calculate cone values. Analysis was performed at two and four degrees of eccentricity from the fovea along the four meridians (nasal, temporal, superior, inferior).

**Results:**

In POAG eyes, the mean ± standard deviation (SD) cone density at 2° considering all meridians was significantly lower than in normal controls (23,058.6 ± 3532.0 cones/mm^2^, and 25,511.7 ± 3157.5 cones/mm^2^, respectively; *p* = 0.003). Cone spacing was 7.3 ± 0.5 µm in POAG and 7.0 ± 0.4 µm in normal controls (*p* = 0.005), and cone regularity was 90.5 ± 4.9% and 93.5 ± 1.9% in POAG and normal controls, respectively (*p* < 0.001). At 4° similar trends were observed. However, no significant differences were found among patients with different severity of POAG (*p* > 0.05).

**Conclusions:**

Using AO Rtx1^TM^, significant differences in retinal photoreceptors mosaic pattern were found between POAG eyes and age-matched controls, indicating a reduction in photoreceptors in POAG. No significant differences in retinal photoreceptor values were found among the three POAG groups.

## Introduction

Glaucoma is a chronic progressive neuropathy that leads to characteristics and morphologic changes in the optic nerve head with the loss of retinal ganglion cells, associated with progressive visual field (VF) loss.

To date, the pathogenesis of primary open-angle glaucoma (POAG) is not fully understood, but various risk factors have been identified including elevated intraocular pressure (IOP), advanced age, high myopia, and a positive family history of glaucoma. However, elevated IOP remains the only demonstrated modifiable risk factor for both the development and progression of glaucoma [1]. Patients with POAG present a progressive thinning of both the retinal nerve fiber layer (RNFL) and the ganglion cell layer (GCL) [2–4]. However, it is unclear whether glaucoma may be associated with retinal photoreceptor damage. Conventional retinal imaging devices do not allow visualization of individual photoreceptors due to higher-order ocular aberrations. This gap could be filled by an innovative technology named adaptive optics (AO), which can eliminate wavefront distortions at the time of examination and allows for the investigation of the microscopic structure of the retina, in vivo.

The primary outcome of the study was to investigate the density, spacing, and regularity of the retinal cones using an AO retinal camera (Rtx1^TM^, Imagine Eyes, Orsay, France) in eyes with POAG and to compare the measurements to those of eyes of healthy age-matched controls. The secondary outcome was to compare retinal cone density, spacing, and regularity between eyes with different severity of POAG.

## Materials and methods

### Study population

Forty-three eyes of twenty-nine patients with POAG and thirty-one eyes of eighteen healthy volunteers were recruited as participants in an ongoing prospective cross-sectional study at the Ophthalmology Unit of the Hospital of Rimini. The study was conducted in adherence to the principles of the Declaration of Helsinki, and written informed consent was obtained from all subjects. Institutional Review Board/Ethics Committee approval was obtained from the local health service of Romagna, Italy (Cod CE: 6754/2023).

The primary objective of the study was to compare cone density, spacing, and regularity between eyes with POAG and healthy age-matched controls, using the Rtx1^TM^ retinal camera AO. The secondary outcome was to compare these measures among eyes with varying degrees of POAG severity.

Each measurement was performed by a single experienced ophthalmologist, who repeated the examination twice in each patient to verify the method’s reproducibility.

All patients in the glaucoma group met POAG diagnostic criteria: elevated IOP (>21 mmHg) history, glaucomatous optic disk appearance, optic nerve head structural changes confirmed by optical coherence tomography (OCT), and corresponding VF defects from the Humphrey visual field test 30-2 (HVF 30-2) performed by an orthoptist. HVF 30-2 test was used to eventually detect temporal wedges peripheral to the blind spot which can occur in glaucoma. Criteria also included a normal anterior segment, absence of clinically significant lens opacities, and an open angle at gonioscopy.

Patients with POAG were categorized into three groups based on VF loss severity, using the Hodapp–Parrish–Anderson (H-P-A) criteria [5, 6], with the most recent VF (less than 3 months) considered. Exclusion criteria encompassed closed-angle, previous retinal detachment, corneal opacities, advanced cataract, age-related macular degeneration, diabetic retinopathy, high myopia (>−6 dioptres), axial length (AL) >26 mm and <21 mm, and false positive or false negative results of ≥20% at HFA. The control group comprised thirty-one healthy subjects selected among hospital workers and patients’ relatives, using the same exclusion criteria.

### Clinical assessment

Each patient underwent a comprehensive ophthalmologic examination, including refraction, best-corrected visual acuity (BCVA), IOP measured with a Goldmann applanation tonometer, gonioscopy, Axial Length (IOLMaster, Carl Zeiss Meditec, Dublin, CA, USA), VF testing using the Humphrey Field Analyzer (HFA; Carl Zeiss Meditec), fundoscopy, and retinal imaging using spectral-domain OCT (Spectralis®, Heidelberg Engineering, Heidelberg, Germany) RNFL, ganglion cells complex (GCC) and AO Rtx1^TM^. Information on medical therapies and comorbidities was collected during the baseline ophthalmic evaluation.

### Retinal imaging with the flood-illuminated AO retinal camera (Rtx1™)

The Rtx1^TM^ is an AO retinal Camera that allows the retina to be examined at a scale where individual photoreceptor cells are visible. Its ultrahigh-resolution images show parafoveal cone photoreceptors as bright spots. Once pharmacological mydriasis was established, AO imaging acquisitions were performed. The subject fixated on an internal target (yellow cross) moved by the operator. The Rtx1^TM^ is based on a Shack–Hartmann wavefront sensor (Haso 32; Imagine Optic, Orsay, France) illuminated at 780 nm and a deformable mirror (mirao 52-e; Imagine Eyes). To ensure image quality, the degree of AO correction, shown on the camera software panel, was checked to be <1 before image acquisition, and the focus was adjusted to optimize cone photoreceptor clarity. A full description of the Rtx1™ has been previously reported [7]. For each eye, a set of forty raw images of the same retinal area was acquired at a rate of 9.5 frames per second, with an exposure time of 10 ms to create a final image of a 4 × 4 degrees field (i.e., 1500 × 1500 pixels) with improved signal-to-noise ratio. Images were acquired at 2° and 4° eccentricity along the four meridians (nasal, temporal, superior, and inferior). For each image, a region of interest (ROI) was defined by the operator in correspondence with an area with the best resolution, avoiding blood vessels where the photoreceptors cannot be counted. After entering the AL of the examined eye, the dedicated software (AOdetect 3.0 SP1; Imagine Eyes) was used for post-processing (segmentation and measurement) of the ROI included in the 4 × 4 degrees field AO image. Cone cells were automatically detected using the manufacturer-provided system software based on peak intensity and diameter. When the algorithm failed to properly label the cones, the ophthalmologist manually labeled the areas where cones were visible but not detected automatically, minimizing potential cone under- or oversampling by the automated software. Cone density was automatically calculated in units of cells/mm^2^ [8], cone spacing in µm, and cone regularity in %. Cone metrics (local density, spacing, and regularity) were assessed at each degree from 2° to 4° eccentricity from the fovea along the four meridians.

**Fig. 1:**
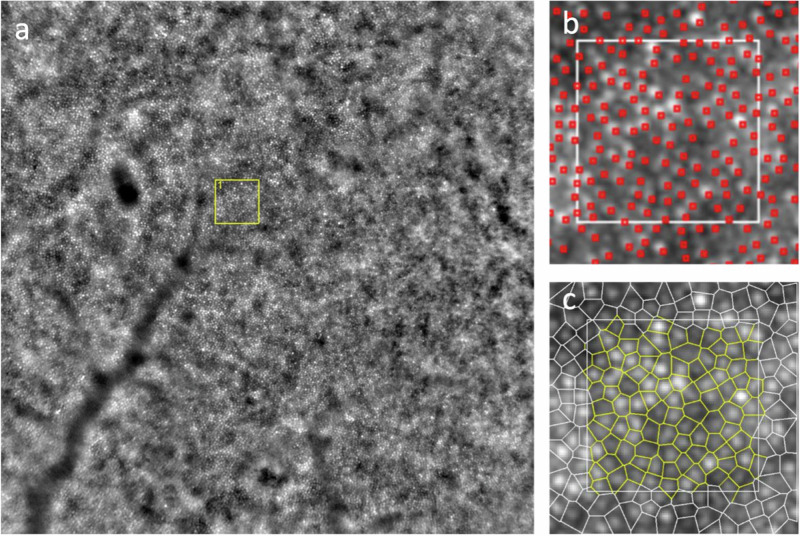
Image of the right eye of a healthy patient focused on the level of photoreceptors in a 63-year-old man at 2° of eccentricity in the temporal quadrant. **a** 4 × 4 degrees field AO image: yellow square represents the ROI; post-processing ROI image (**b**) cone density and (**c**) spatial distribution of cones.

**Fig. 2:**
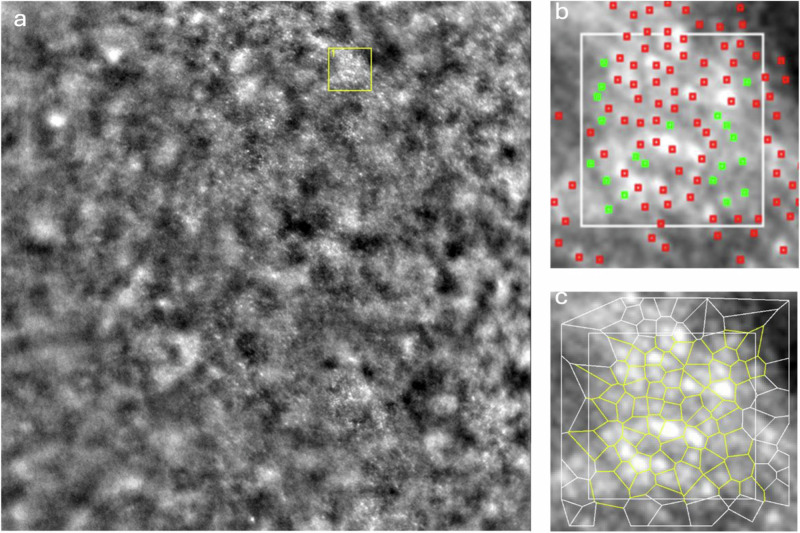
Image of the left eye of a glaucomatous patient focused on the level of photoreceptors in a 66-year-old man at 2° of eccentricity in the nasal quadrant. **a** 4 × 4 degrees field AO image: yellow square represents the ROI; post-processing ROI image (**b**) cone density and (**c**) spatial distribution of cones.

A representative image of the right eye of a 63-year-old healthy control focused at 2° of eccentricity in the temporal quadrant is depicted in Fig. 1. Figure 2 shows the left eye of a glaucomatous patient focused on the level of photoreceptors in a 66-year-old man at 2° of eccentricity in the nasal quadrant.

### Statistical analysis

Statistical analyses were performed using STATA/IC 17 (StataCorp LP, College Station, TX, USA) and SPSS Statistics (version 27. IBM Corp., Chicago, IL). Continuous variables were reported as the median and interquartile range (IQR) and were compared by means of the Mann–Whitney U-test and Kruskal–Wallis test. Categorical variables were reported as frequencies and percentages and were compared with Fisher exact test.

In order to account for the correlation between the eyes of the same subject, differences in cone density, spacing, and regularity between case and control participants were evaluated using generalized estimating equations. For all tests, a 2-sided *p*-value < 0.05 was required for statistical significance.

A sample size of thirty pairs with a correlation of 0.500 achieves 80% power to detect a difference of 1055 cones/mm^2^ in terms of density between the glaucomatous and the healthy eyes at the 0.050 significance level (alpha) using a two-sided Wilcoxon Signed-Rank test (31).

## Results

### Demographic characteristics

We included seventy-four eyes from forty-seven participants in this study, comprising forty-three eyes from twenty-nine POAG patients (sixteen eyes with mild glaucoma, fifteen eyes with moderate glaucoma, and twelve eyes with severe glaucoma) and thirty-one eyes from eighteen healthy control subjects. Demographic data for the POAG patients and the control group are presented in Table 1. The demographic data of POAG patients were comparable to those of the control group (*p* > 0.05). In the POAG group, thirty-nine eyes received treatment with topical antiglaucoma medications: twelve eyes (30.7%) with one medication, 9 (23%) with two medications, 14 (35.8%) with three medications, and 4 (10.2%) with four medications. Cataract surgery was performed in 7 eyes (16.3%) of POAG group and 4 eyes of healthy control subjects (12.9%). Moreover, sixteen eyes (37.2%) of POAG group underwent IOP-lowering surgery. The median MD was −3.2 (IQR = −4.1, −0.9) in the mild glaucoma group, −9.8 (IQR = −11.4, −7.2) in the moderate glaucoma group, and −18.8 (IQR = −26.7, −13.1) in the severe glaucoma group, significantly lower than in the healthy control subjects, where MD ranged from −1 to 1 (*p* < 0.05). See Table 2. The median spherical equivalent (SE) (dioptres) was –0.8 (IQR = –2.3, –0.0) in the mild glaucoma group, 0.0 (IQR = −1.0, 1.0) in the moderate glaucoma group, and –0.5 (IQR = −1.0, 0.3) in the severe glaucoma group, while SE was 1.0 (IQR = −0.5, 2.3) in the healthy control subjects.

**Table 1:** Comparison between demographic data of primary open-angle glaucoma (POAG) and healthy subjects.

	POAG (*n* = 43 eyes)	Control (*n* = 31 eyes)	*p* value
Age	66 (60–74)	66 (63–70)	0.625
Sex, male/female (%)	16/13 (55.1%;44.9%)	10/8 (55.5%; 44.5%)	0.873
BCVA (LogMAR)	0.10 (0.00–0.20)	0.00 (0.00–0.00)	0.453
Axial length (mm)	23.7 (22.7–24.7)	23.4 (22.8–23.9)	0.339
IOP (mmHg)	15 (13–17)	14 (13–17)	0.454
RNFL G	54 (46–74)	96 (93–100)	**<0.001**
GCC G	33 (28–39)	52 (49–54)	**<0.001**

Data are expressed as median and interquartile range IQR.

*BCVA* best correct visual acuity, *logMAR* logarithm of the minimum angle of resolution, *IOP* intraocular pressure, *RNFL* retinal nerve fiber layer, *GCC* ganglion cell complex.

Statistically significant *p*-values are bold.

**Table 2:** Media and Deviation standard of Visual field values in mild, moderate and severe primary open-angle glaucoma (POAG) subjects.

	Mean deviation (Media ± DS)	Pattern standard deviation (Media ± DS)
Mild POAG	−2.69 ± 1.91	3.98 ± 1.76
Moderate POAG	−9.36 ± 2	10.02 ± 3.44
Severe POAG	−19.84 ± 6.52	9.62 ± 3.43

*MD* mean deviation, *PSD* pattern standard deviation.

### Photoreceptor parameters using Rtx1 ^TM^

We analyzed the photoreceptor mosaic pattern at 2° and 4° eccentricity from the fovea along the four meridians (nasal, temporal, superior, inferior) in both healthy and POAG eyes to compare photoreceptor parameters. The average values for cone density, spacing, and regularity between glaucomatous eyes and age-matched controls are shown in Supplement 1. We manually labeled the areas where cones were visible but not detected automatically in the 8.1% (6 eyes).

The percentage of density reduction between the glaucoma group and healthy controls was 9%, 5%, 11%, 11%, 11%, 1%, 9%, and 5% at 2 nasal, 4 nasal, 2 temporal, 4 temporal, 2 superior, 4 superior, 2 inferior, 4 inferior, respectively. Additionally, we examined the differences in density, spacing, and regularity between patients with different severities of POAG according to MD. See Supplement 2.

Considering all meridians, the overall average density, spacing, and regularity values at 2° and 4° were significantly different between the POAG group and the healthy control group. See Table 3. While there was no significant difference among mild, moderate, and severe glaucoma.

**Table 3:** Comparison between glaucomatous groups and healthy control groups in Terms of cone density, regularity, and spatial organization (mean ± standard deviation), considering all meridians.

	Glaucoma group (*n* = 43 eyes)	Control group (*n* = 31 eyes)	*p* value
Density (cells/mm^2^)			
2°	23,058.6 ± 3532.0	25,511.7 ± 3157.5	**0.003**
4°	20,017.8 ± 3105.2	21,298.5 ± 2491.2	**0.011**
Spacing (micron)			
2°	7.3 ± 0.5	7.0 ± 0.4	**0.005**
4°	7.9 ± 0.5	7.6 ± 0.4	**0.016**
Regularity (%)			
2°	90.5 ± 4.9	93.5 ± 1.9	**<0.001**
4°	91.4 ± 4.4	94.1 ± 2.4	**0.001**

Statistically significant *p*-values are bold.

## Discussion

Glaucoma is known to cause damage to the inner retina [2–4]. In recent years, attention has shifted to the involvement of the outer retina, particularly photoreceptor damage. However, the limited resolution of conventional retinal imaging instruments did not allow for a direct assessment of the structural integrity of the cone cells. This became possible with the development of new technologies such as AO. By applying AO to various retinal imaging modalities, it is now possible to visualize individual photoreceptors in vivo. Rtx1^TM^ is an AO retinal camera that can be used to visualize high-resolution images of retinal microstructures in vivo [9–14].

The most important finding of our study was the demonstration of a statistically significant difference in the photoreceptor mosaic pattern between eyes with POAG and an age-matched population of healthy patients (*p* < 0.05).

Photoreceptor damage in acute hypertensive glaucoma is well established, but there is a lack of evidence in patients with chronic glaucoma [15–18].

Bui et al. demonstrated primary photoreceptor damage after acute ocular hypertension in an animal model [15]. Panda et al. reported that eyes with secondary angle-closure glaucoma had fewer photoreceptor cells than eyes in the non-glaucomatous control group [16].

Nork’s studies, based on histopathologic findings, were among the first to investigate outer retinal involvement [19].

The initial hypothesis of Nork et al. was that abnormalities in color vision and electrophysiologic effects in POAG patients might be related to photoreceptor cell changes. In their study, they histologically examined one hundred twenty-eight eyes from sixty-four donors clinically diagnosed with POAG and ninty control eyes, finding that the cone nuclei in the outer part of the outer nuclear layer (ONL) were enlarged and the bodies were swollen in the POAG group compared to controls [19]. They suggested three possible explanations. Because histologic evidence of photoreceptor enlargement occurs early in the disease history, this effect could be part of the normal course of glaucoma and occur independently of ganglion cell damage. Our results could support this explanation because we did not find significant differences in the photoreceptor mosaic pattern among mild, moderate, and severe glaucoma (*p* > 0.05). The second hypothesis is that photoreceptor damage could be a consequence of ganglion cell death. Third, photoreceptor swelling could be the result of ischemic damage due to a reduction in choroidal blood flow, secondary to increased IOP. Photoreceptor damage leads to a decrease in the reuptake of glutamate, the major excitatory neurotransmitter of photoreceptors, and thus to its accumulation. The accumulation of glutamate would, in turn, lead to the overexcitation of ganglion cells, triggering their apoptosis. This theory is supported by evidence that ganglion cells die by apoptosis in glaucoma, and that eyes with POAG have increased glutamate levels in the vitreous [19].

Other studies have hypothesized photoreceptor involvement in eyes with POAG based on color vision tests and electrophysiologic studies that revealed a pattern of color vision loss suggestive of cone damage [20, 21].

According to Koellner’s rule, diseases of the optic nerve usually cause red-green color defects, while diseases affecting the outer retina cause blue-yellow defects. Patients with glaucoma, however, usually show a blue-yellow color defect [21]. This fact suggests that the color vision defect in glaucoma is consistent with a retinal degeneration process rather than one that affects only the optic nerve. Schneck and Haegerstrom-Portnoy [22] and Menage et al. [23] reported that patients with optic neuritis have mixed blue-yellow and red-green color defects, rather than the expected red-green defects. This observation highlights the involvement of both the inner and outer retina in optic neuropathies [24].

Fan et al. measured and compared photoreceptor layer thickness between normal and glaucomatous eyes using OCT. They concluded that glaucomatous damage may be associated with a structural change in the photoreceptor layer. The major limitation of their study was the inability to assess individual photoreceptors with OCT, and they therefore used photoreceptor layer thickness measurement as a surrogate [25]. Recently, Choi et al. utilized three ultrahigh-resolution retinal imaging systems to investigate the integrity of the inner and outer retinal layers in patients with different types of optic neuropathy and VF defects, including AO technology. Structural changes in photoreceptors were found when the overlying inner retinal layers were permanently damaged, whereas the mosaic pattern of photoreceptors was normal in patients with transient VF defects [24, 26]. They also observed dark areas in the cone mosaic of glaucomatous eyes using an AO fundus camera. The hypothesis was that these areas contained cones that were rendered invisible by changes in their waveguide properties, thus damaging the photoreceptor. Indeed, the extent of the dark areas was related to the VF defect [24]. Consistent with these findings, Werner et al. compared subjects with nonglaucomatous and glaucomatous optic neuropathy and concluded that photoreceptor damage was present in all cases of long-term VF loss. In cases with transient VF impairment and a normal inner retina, no abnormalities were found in the outer retina [27]. Photoreceptor involvement in glaucoma is also supported by functional electroretinogram studies (ERG) [28, 29]. Vaegan et al. demonstrated a reduction and delay in ERG a and b waves in glaucoma patients, comparable to those observed in early cone-rod dystrophy [28]. On the other hand, several studies did not report a correlation between glaucoma and photoreceptor damage. Kendell et al. showed histologically that photoreceptor density and ONL thickness did not change between eyes with POAG and control subjects [30]. Hasegawa and colleagues demonstrated the integrity of cones in glaucoma eyes using prototype AO Scanning Laser Ophthalmoscopy (AO-SLO) [31]. They supported the hypothesis of retrograde trans-synaptic degeneration [32]. They enrolled glaucoma patients who had experienced parafoveal VF loss at least 3 years previously. Additionally, they examined the photoreceptors at the corresponding sites of VF defect. They found no difference between glaucoma patients and the control group in terms of cone density and spatial organization of cones [31], even in the retinal areas corresponding to the VF defect examined with the HVF test. Another result of our study was that there were no significant differences in the photoreceptor mosaic pattern between patients with different levels of POAG severity (*p* > 0.05), classified by VF defect tested with the HVF test. Fan and coworkers reported that the foveal ONL was thicker in eyes with early-stage glaucoma than in healthy eyes, whereas there was no difference between healthy and moderately-to-advanced glaucomatous eyes [25]. This thickening of the ONL is consistent with the postmortem study by Nork and colleagues [19]. This may suggest that photoreceptors are swollen in the early stages of glaucoma, whereas no photoreceptor changes occur in the advanced stages or areas with RNFL defects. This finding is consistent with our study, in which we found a difference in the mosaic pattern between glaucomatous and healthy eyes, but no difference between mild, moderate, and advanced POAG. Furthermore, in agreement with previous studies, we showed that photoreceptor cells decrease with increasing distance from the fovea along the four meridians [33].

Among the strengths of our study, each measurement was performed by an ophthalmologist (ET) who repeated the examination twice in each patient and compared the results of the two examinations to verify the reproducibility of the method. Many studies have confirmed that age and AL are factors that influence cone density. In our study, we included patients and healthy subjects with similar ages (*p* > 0.05) and AL < 26 mm, to avoid the presence of confounding factors. Since Rtx1^TM^ allows the input of the exact AL of the studied eye to obtain an accurate measure of the photoreceptor mosaic pattern, we entered the exact AL of the eye to be more accurate. In addition, we decided to include 8 regions of interest (2° and 4° eccentricity from the fovea along the four meridians nasal, temporal, superior, and inferior) to cover a larger area of the retina and not limit the analysis to the foveal region.

The main limitation of our study is the small patient cohort. Second, the exclusion of high myopia and the lack of ethnic diversity do not permit the generalizability of the results. Third, we manually selected the area of the image to be analyzed, which may be influenced by the operator. In addition, an experienced and qualified ophthalmologist is required to acquire and process the image.

Our future goal is to increase the number of POAG patients to confirm our results and eventually clarify the role of the outer retina in glaucoma.

## Conclusion

In this study, we observed a reduction in photoreceptor density, and regularity, and a consequent increase in spacing, in individuals with POAG compared to healthy age-matched controls. Interestingly, there were no significant differences in retinal photoreceptor values among the three groups of POAG patients, categorized by the severity of the field defect suggesting an early involvement of photoreceptors. To the best of our knowledge, this is the first investigation exploring disparities in parafoveal photoreceptor density, regularity, and spacing across varying degrees of POAG severity. This is only a first step toward a complete understanding of the pathogenesis of glaucoma. Future studies are needed to understand the photoreceptor involvement in POAG patients.

Supplemental material is available at Eye’s website

## Summary

### What was known before

- Glaucoma is known to cause damage to the inner retina, but it is unclear whether it is associated with damage to retinal photoreceptors.
- The limited resolution of conventional retinal imaging instruments did not allow for a direct assessment of the structural integrity of cone cells.
- Rtx1 AO retinal camera permits the visualization of high-resolution images of retinal microstructures in vivo.

### What this study adds

- Rtx1 AO retinal camera permits the observation of a statistically significant difference in the photoreceptor mosaic pattern between eyes with POAG and an age-matched population of healthy patients.
- No significant differences in retinal photoreceptor values were found among patients with different degrees of glaucomatous damage, suggesting early involvement of photoreceptors.
- This study represents the first investigation to explore disparities in parafoveal photoreceptor density, regularity, and spacing across varying degrees of POAG severity.

## Supplementary information

Supplement 1

Supplement 2

## Data Availability

The datasets used and/or analysed during the current study are available from the corresponding author on reasonable request.
