# Supplementary material for: A parafoveal retinal cones analysis using adaptive-optics retinal camera in patients with primary open angle glaucoma
Source: Eye (Lond). 2024 Sep 2;38(15):2932–8. doi: 10.1038/s41433-024-03191-1 (PMC11461485; doi:10.1038/s41433-024-03191-1)
Supplement: Supplementary file 1 — Supplement 1 [file 41433_2024_3191_MOESM1_ESM.docx]

| **Density (cells/mm^2^)** | **Glaucoma group**  **(n= 43 eyes)** | **Control group**  **(n= 31 eyes)** | **Univariate GEE** | | **p value** |
| --- | --- | --- | --- | --- | --- |
|  |  |  | **β** | **95% CI** |  |
|  |  |  |  |  |  |
| 2n | 23 438.6 ± 3 494.7 | 25 751.2± 3 037.3 | -2 312.5 | -4 114.7  -510.4 | **0.012** |
| 4n | 20 915.0±3 273.0 | 22 003.5±3 226.5 | -1 088.5 | -2 802.7  625.6 | 0.213 |
| 2t | 23 229.9±4 060.9 | 26 204.5±3 301.3 | -2 974.6 | -5 102.9  -846.2 | **0.006** |
| 4t | 20 545.5±3 575.0 | 23 123.0±3 117.9 | -2 577.5 | -4 449.7  -705.4 | **0.007** |
| 2s | 22 533.0 ± 3 978.5 | 25 215.0±4 142.0 | -2 682.0 | -4 925.7  -438.3 | **0.019** |
| 4s | 19 375.0±3 673.8 | 19 642.0±2 051.5 | -267.0 | -1 672.9  1 138.8 | 0.710 |
| 2i | 23 158.4 ± 4 373.2 | 25 567.3 ±3 489.5 | -2 408.9 | -4 515.7  -302.1 | **0.025** |
| 4i | 19 441.9±3 307.1 | 20 425.5±3 657.6 | -983.6 | -2 927.9  960.7 | 0.321 |
| **Spacing (micron)** | **Glaucoma group**  **(n= 43 eyes)** | **Control group**  **(n= 31 eyes)** | **Univariate GEE** | | **p value** |
|  |  |  | **β** | **95% IC** |  |
|  |  |  |  |  |  |
| 2n | 7.2 ± 0.5 | 6.9±0.4 | 0.3 | 0.1  0.6 | **0.013** |
| 4n | 7.7±0.6 | 7.5±0.5 | 0.2 | -0.1  0.5 | 0.179 |
| 2t | 7.3±0.6 | 6.9±0.5 | 0.4 | 0.1  0.7 | **0.004** |
| 4t | 7.8±0.6 | 7.3±0.5 | 0.4 | 0.1  0.7 | **0.003** |
| 2s | 7.4±0.6 | 7.0±0.6 | 0.4 | 0.1  0.7 | **0.016** |
| 4s | 8.0±0.7 | 7.9±0.4 | 0.1 | -0.2  0.4 | 0.470 |
| 2i | 7.3 ±0.6 | 6.9±0.5 | 0.4 | 0.1  0.7 | **0.017** |
| 4i | 8.0±0.6 | 7.8±0.7 | 0.2 | -0.2  0.5 | 0.318 |
| **Regularity (%)** | **Glaucoma group**  **(n= 43 eyes)** | **Control group**  **(n= 31 eyes)** | **Univariate GEE** | | **p value** |
|  |  |  | **β** | **95% IC** |  |
| 2n | 88.9 ± 13.8 | 93.3±3.6 | -4.4 | -8.8  0.1 | 0.054 |
| 4n | 91.5±5.7 | 94.4±3.0 | -3.0 | -5.1  -0.8 | **0.007** |
| 2t | 90.2±4.8 | 92.6±3.5 | -2.4 | -4.4  -0.5 | **0.014** |
| 4t | 92.8±5.8 | 94.8±3.1 | -2.1 | -4.2  0.1 | 0.065 |
| 2s | 91.2 ±5.7 | 94.5±2.6 | -3.4 | -5.3  -1.5 | **0.001** |
| 4s | 90.2±7.1 | 93.7±4.1 | -3.5 | -5.9  -1.1 | **0.004** |
| 2i | 92.0 ±4.4 | 93.8±3.9 | -1.8 | -3.5  -0.0 | **0.048** |
| 4i | 91.6±4.9 | 93.5±4.6 | -1.9 | -4.2  0.4 | 0.105 |

**Supplement 1**. Comparison Between Glaucomatous and Healthy Eyes in Terms of Cone Density, Regularity, and Spatial Organization (mean ± standard deviation) and results of univariate GEE analysis. GEE= generalized estimating equations. CI= confidence interval; 2n= 2° nasal; 4n= 4° nasal; 2t= 2° temporal; 4t= 4° temporal; 2s= 2° superior; 4s= 4° superior; 2i= 2° inferior; 4i= 4° inferior.
