# Supplementary material for: A parafoveal retinal cones analysis using adaptive-optics retinal camera in patients with primary open angle glaucoma
Source: Eye (Lond). 2024 Sep 2;38(15):2932–8. doi: 10.1038/s41433-024-03191-1 (PMC11461485; doi:10.1038/s41433-024-03191-1)
Supplement: Supplementary file 2 — Supplement 2 [file 41433_2024_3191_MOESM2_ESM.docx]

| **Density (cells/mm2)** | **Mild glaucoma**  **(n=16 eyes)** | **Moderate glaucoma (n= 15 eyes)** | **Severe glaucoma (n= 12 eyes)** | **Univariate GEE between Mild and Moderate glaucoma** | | **p value** | **Univariate GEE between Mild and Severe glaucoma** | | **p value** |
| --- | --- | --- | --- | --- | --- | --- | --- | --- | --- |
|  |  |  |  | **β** | **95% CI** |  | **β** | **95% CI** |  |
| **2n** | 23 991.6 ±  3 401.6 | 23 115.8 ±  3 278.2 | 23 024.3 ±  4 003.6 | 967.3 | -1 451.5,  3 386.1 | 0.433 | 91.5 | -2 542.7,  2 725.7 | 0.946 |
| **4n** | 20 488.9±  2 839.7 | 21 343.4±  3 847.3 | 21 028.5±  3 372.9 | -539.7 | -2 646.0,  1 566.6 | 0.616 | 314.8 | -2 102.6,  2 732.2 | 0.799 |
| **2t** | 23 035.1 ±  4 429.9 | 22 991.1 ±  3 620.4 | 23 788.3±  4 364.6 | -753.3 | -3 630.5,  2 124.0 | 0.608 | -797.3 | -3 691.8,  2 097.3 | 0.589 |
| **4t** | 19 347.4±  2 942.1 | 21 495.8±  4 244.5 | 20 955.0±  3 250.2 | -1 607.6 | -3 830.9,  615.6 | 0.156 | 541.0 | -2 107.4,  3 189.0 | 0.689 |
| **2s** | 22 868.3 ±  3 996.7 | 22 446.1 ±  4 364.9 | 22 194.5±  3 755.9 | 673.8 | -1 966.3,  3 314.0 | 0.617 | 251.6 | -2 770.4,  3 273.7 | 0.870 |
| **4s** | 18 626.7±  2 804.3 | 19 750.9±  4 661.4 | 19 902.8±  3 441.3 | -1 276.1 | -3 557.1,  1 004.9 | 0.273 | -151.8 | -3 001.0,  2 697.4 | 0.917 |
| **2i** | 22 317.1 ±  4 107.0 | 23 180.4 ±  4 868.5 | 24 256.5 ±  4 282.6 | -1 939.4 | -53 40.9,  1 462.0 | 0.264 | -1 076.1 | -4 294.3,  2 142.1 | 0.512 |
| **4i** | 19 040.2±  3 103.9 | 20 175.3±  4 131.1 | 19 060.9±  2 402.9 | -20.7 | -1 897.1,  1 855.6 | 0.938 | 1 114.4 | -1 223.2,  3 451.9 | 0.350 |
| **Spacing (micron)** | **Mild glaucoma**  **(n=16 eyes)** | **Moderate glaucoma (n= 15 eyes)** | **Severe glaucoma (n= 12 eyes)** | **Univariate GEE between Mild and Moderate glaucoma** | | **p value** | **Univariate GEE between Mild and Severe glaucoma** | | **p value** |
|  |  |  |  | **β** | **95% IC** |  | **β** | **95% IC** |  |
| **2n** | 7.2 ± 0.5 | 7.3 ± 0.5 | 7.3 ± 0.6 | -0.1 | -0.5,  0.2 | 0.496 | 0.0 | -0.4,  0.4 | 0.976 |
| **4n** | 7.8±0.5 | 7.6±0.7 | 7.7±0.6 | 0.1 | -0.3,  0.5 | 0.604 | -0.0 | -0.4,  0.4 | 0.897 |
| **2t** | 7.3±0.7 | 7.3±0.5 | 7.2±0.6 | 0.1 | -0.3,  0.6 | 0.550 | 0.1 | -0.3,  0.5 | 0.562 |
| **4t** | 8.0±0.6 | 7.6±0.7 | 7.7±0.5 | 0.3 | -0.1,  0.7 | 0.112 | -0.1 | -0.5,  0.4 | 0.817 |
| **2s** | 7.4 ±0.7 | 7.4 ±0.7 | 7.5 ±0.6 | -0.1 | -0.5,  0.4 | 0.690 | -0.0 | -0.5,  0.4 | 0.871 |
| **4s** | 8.1±0.6 | 8.0±0.8 | 7.8±0.6 | 0.3 | -0.1,  0.7 | 0.175 | 0.1 | -0.4,  0.6 | 0.615 |
| **2i** | 7.4 ±0.6 | 7.3 ± 0.7 | 7.2 ± 0.6 | 0.3 | -0.2,  0.8 | 0.245 | 0.2 | -0.3,  0.6 | 0.501 |
| **4i** | 8.1±0.6 | 7.8±0.7 | 8.0±0.5 | 0.1 | -0.3,  0.4 | 0.730 | -0.2 | -0.6,  0.2 | 0.418 |
| **Regularity (%)** | **Mild glaucoma**  **(n= 16 eyes)** | **Moderate glaucoma (n= 15 eyes)** | **Severe glaucoma (n= 12 eyes)** | **Univariate GEE**  **between Mild and Moderate glaucoma** | | **p value** | **Univariate GEE**  **between Mild and severe glaucoma** | | **p value** |
|  |  |  |  | **β** | **95% CI** |  | **β** | **95% CI** |  |
| **2n** | 84.8 ± 21.3 | 92.2 ± 2.4 | 91.2 ± 3.8 | -6.4 | -16.8,  4.1 | 0.232 | 1.1 | -0.3,  2.4 | 0.121 |
| **4n** | 89.9±7.1 | 91.8±3.5 | 93.3±5.4 | -3.4 | -8.3,  1.6 | 0.187 | -1.5 | -4.8,  1.8 | 0.378 |
| **2t** | 88.9±5.2 | 91.1±4.7 | 90.9±4.1 | -2.1 | -5.5,  1.4 | 0.239 | 0.1 | -2.9,  3.1 | 0.928 |
| **4t** | 92.4±5.1 | 91.0±7.2 | 95.5±3.5 | -3.1 | -6.3,  0.1 | 0.058 | -4.5 | -8.7,  -0.2 | 0.038 |
| **2s** | 92.1 ± 4.4 | 89.5 ± 6.9 | 92.1 ± 5.7 | 0.0 | -3.4,  3.4 | 0.991 | -2.6 | -6.7,  1.4 | 0.206 |
| **4s** | 89.1±7.4 | 90.5±8.7 | 91.2±4.2 | -2.1 | -5.9,  1.6 | 0.266 | -0.7 | -4.6,  3.1 | 0.711 |
| **2i** | 92.3 ±4.6 | 91.8 ± 4.7 | 92.0 ± 4.3 | 0.3 | -3.0,  3.6 | 0.839 | -0.1 | -3.7,  3.5 | 0.938 |
| **4i** | 90.6±6.3 | 91.3±3.5 | 93.2±4.3 | -2.6 | -6.5,  1.2 | 0.180 | -1.9 | -5.0,  1.3 | 0.239 |

**Supplement 2.** Comparison Between 3 Glaucomatous groups in relation to the severity of the field defect in Terms of Cone Density, Regularity, and Spatial Organization (mean ± standard deviation) and results of univariate GEE analysis. GEE= generalized estimating equations; CI= confidence interval; 2n= 2° nasal; 4n= 4° nasal; 2t= 2° temporal; 4t= 4° temporal; 2s= 2° superior; 4s= 4° superior; 2i= 2° inferior; 4i= 4° inferior.
